# Impact of water deficit on the development and senescence of tomato roots grown under various soil textures of Shaanxi, China

**DOI:** 10.1186/s12870-021-03018-1

**Published:** 2021-05-28

**Authors:** Husain Ahmad, Jianming Li

**Affiliations:** College of Horticulture, Northwest A&F University, Yangling, 712100 Shaanxi China

**Keywords:** Soil texture, Root anatomy, Water deficit, Endogenous hormones

## Abstract

**Purpose:**

Water scarcity is expected to extend to more regions of the world and represents an alarming threat to food security worldwide. Under such circumstances, water holding capacity is an important agronomic trait, which is primarily controlled by soil texture.

**Methods:**

Our work examined three different soil textures from three cities of Shaanxi Province in China, i.e., silt-sandy loam from Yulin (north of Shaanxi), loam—clay loam from Yangling (middle and western part of Shaanxi), and clay loam-clay from Hanzhong soil (south of Shaanxi), at two moisture levels, i.e., field capacity of 70–75% (well-watered) and 50–55% (water deficit).

**Results:**

The differences in soil particle sizes altered the soil physiochemical properties and soil enzymatic activities. Soil urease and ß-glucosidase activities were significantly higher in the Yangling soil under the well-watered treatment, while the differences were nonsignificant under the water deficit conditions. The leaf photosynthesis rate and total chlorophyll content were significantly higher in Hanzhong soil after 15 days of treatment; however, the overall highest plant length, root cortex diameter, and xylem element abundance were significantly higher in Yangling soil under the water deficit conditions. Furthermore, comparable differences were observed in antioxidant defence enzymes and endogenous hormones after every 15 days of treatments. The auxin, gibberellic acid and cytokinin concentrations in leaves and roots were comparably high in Yangling soil, while the abscisic acid concentrations were higher in Hanzhong soil under the water deficit conditions.

**Conclusions:**

Our findings concluded that soil compaction has a significant role not only in root morphology, growth, and development but also in the soil physicochemical properties and nutrient cycle, which are useful for the growth and development of tomato plants.

## Background

Limited water availability is expected to extend to more regions due to growing food demands and increasing freshwater scarcity [1]. Water scarcity is one of the major threats to agriculture productivity and causes dramatic changes in the plant physiology, biochemical and cellular activities of most plants, i.e., photosynthesis, respiration, transpiration, and hormone imbalance [2]. One of the initial responses of plants to water deficit is the formation of reactive oxygen species (ROS), including hydrogen peroxide (H_2_O_2_) and oxygen radical (O_2_^−^). ROS are normally produced in plants and represent intrinsic regulators of cell cycle progression; however, their overproduction disrupts growth and developmental functions by interacting with nucleic acids, lipids, membrane structures, etc. and eventually leads to plant cell death (PCD) [3]. Water deficit conditions dehydrate plant tissues and cause an imbalance of root water uptake and leaf transpiration. According to [4], plant water status is the first signal that induces plants to respond to stress conditions.

Plants have developed sophisticated mechanisms of defence with immediate responses to stress conditions, among which the plant enzymatic antioxidant defence systems play an important role in enhancing tolerance to plants [5]. Roots are the primary organs in plants through which they absorb water and nutrients from the soil and act as sensors for water deficit conditions [6]. The root apical meristem consists of rapidly dividing cells, and its establishment is altered under drought conditions. Studies have proven that the balance of the redox status of the roots is important for cell division and multiplication in the root apical meristem or that it may interfere with the biosynthesis and transport of hormones, especially auxin. The redox balance is mainly dependent upon the activity of antioxidant enzymes [7, 8], particularly the ascorbate–glutathione cycle [9]. Plant endogenous hormones also have a key role in plant defence by mediating growth and development and improving source sink relationships in varying environments [10]. Abscisic acid (ABA) in plants plays a pivotal role in drought resistance, and it can effectively regulate stomatal closure, reduce transpiration, increase the activity of the antioxidant system and improve the efficiency of ROS scavenging [11, 12]. The concentrations of gibberellic acid (GA_3_), cytokinin (CTK), and auxin (IAA) were increased with the higher activity of antioxidant defence enzymes in plants [13]. The decrease in GA_3_ reduces cell division and differentiation, while lower CTK levels cause senescence in the whole plant. Moreover, evidence shows that ABA crosstalk occurs with other hormones at the transcriptional level, which influences plant physiological responses to stress conditions [14].

In plants, the most complicated process is the mechanism of assimilation of photosynthates in sink organs [15]. Studies have proven that plant growth and productivity are markedly reduced by reduced photosynthetic activity (source); however, it is now evident that source activity is also dependent upon sink demand. The imbalance between sink-source relationships results in alterations of source photosynthetic activity per unit leaf area, which will affect its potential, even under favourable conditions [16, 17]. Plant roots develop according to the available volume and environment, and they affect the plant’s physiological and chemical processes to establish appropriate growth [18]. Recently, the relationship among soil water content, soil nutrition, and root growth has gained much attention because inadequate soil moisture reduces nutrient accessibility and affects root architecture [19]. The root system functions by penetrating the primary root deep in the soil and extending lateral roots to explore the soil for water and nutrients; however, it is significantly dependent upon soil physio-chemical properties and soil moisture content [20, 21].

The water holding capacity of a soil is a very important agronomic characteristic that play a key role in sustaining plant growth and development [22]. Water holding capacity is controlled primarily by the soil particle size and the soil organic matter content. Soils that hold substantial amounts of water are less subjected to nutrient leaching losses. Soil texture is based on the percentage of sand, silt, and clay particles, and small-sized particles (silt and clay) have a larger surface area compared to larger sand particles, which allow the soil to hold a greater amount of water. Studies have revealed that soils with a higher percentage of clay particles have a higher bulk density, which restricts root growth and development; moreover, it has a major impact on several soil attributes, including water availability, nutrients, soil porosity, and soil metabolic status, and has affected millions of hectares globally [23, 24]. The metabolic status of a soil can be expressed by the transformation and decomposition of organic matter and synthesis of humus. The recycling of debris from plants and microbes consists of a myriad of complex biochemical reactions through various enzymatic and catalytic processes and is highly correlated with the soil particle size and soil organic matter content [25, 26]. The texture of Shaanxi Province in northwestern China greatly varies from silt-sandy loam (Yulin, north of Shaanxi) to loam-clay loam (Yangling, the middle and western part of Shaanxi) and clay loam-clay (Hanzhong, south of Shaanxi) [27]. Tomato plants can be grown in these soils; however, a loam texture is preferred due to the higher air exchange and efficient drainage. In several studies, much focus has been placed on the mechanisms of sink signalling through alterations in source activity. However, the role of soil texture on root growth and development under limited water availability and hormonal crosstalk have not been investigated in horticulture crops. Therefore, our main objectives were to observe tomato root growth and development, root architecture system, senescence, and hormonal crosstalk under the water deficit conditions in different soil textures of Shaanxi, P.R. China.

## Methods

### Soil and plant materials

An experiment was conducted at Northwest A&F University, Yangling, Shaanxi, China, at a greenhouse facility using tomato (*Solanum lycopersicum* L.) Cv Jinpeng No 1 (bought from Yufeng seeds company, Yangling, China) to investigate the root and shoot responses under two water regimes and three different soil textures. The soil was selected from agricultural productive land in the north, middle west, and southern parts of Shaanxi Province (northwestern province) of P.R. China by obtaining a proper permission from the respective authorities. The soil texture in these areas varies from silt-sandy loam (Yulin, north of Shaanxi) to loam-clay loam (Yangling, the middle and western parts of Shaanxi) and clay loam-clay (Hanzhong, south of Shaanxi). The soil from each area was individually mixed, and organic matter and fertilizer were added before the pots were filled. The soil was then transferred to pots (30 cm diameter), and a total of 20 pots were filled per treatment in a single replication. To obtain homogenous plants, tomato seeds were grown in plastic trays with the respective soil used as a growth medium and later transplanted into pots inside a glass house at an average daily temperature of 27/22 °C and 70% humidity. The physico-chemical properties of soil are presented in Table 1.

**Table 1:** Physico-chemical properties of Hanzhong, Yulin, and Yangling soils

Physico-chemical properties of soil	Hanzhong	Yulin	Yangling
Clay (< 0.002 mm) %	39.15	4.64	27.87
Silt (0.05–0.002 mm) %	31.48	88.50	56.13
Sand (> 0.05 mm) %	29.37	6.98	16
pH (1:2.5)	8.4	8.1	8.7
EC (1:2.5) (µS cm^−1^)	317	186	259
Total organic carbon g kg^−1^	7.9	7.5	8.2
Total Nitrogen g kg^−1^	1	0.7	0.9
Total phosphorus g kg^−1^	18.2	16.8	18.9
Total potassium g kg^−1^	241	229	249

### Water treatments

The field capacity for different soil textures was tested gravimetrically as described by [28], and every pot was irrigated to its field capacity. After twenty-five days, plants were irrigated under two water regimes: (1) well-watered conditions, i.e., irrigation according to 70–75% of the field capacity; and (2) water-deficient conditions, i.e., 50–55% of the respective field capacity. Field capacity was achieved by weighing pots three times a week and irrigating with the required water to maintain the field capacity in both watering regimes.

### Plant growth and photosynthetic pigments

Plant growth and chlorophyll contents were determined at the end of the experiment. Shoot length was determined by using a measuring tape, while the chlorophyll a and b and carotenoid contents were determined according to Arnon, 1949 [29]. Fresh tomato leaves (0.3 g) were extracted in 96% ethanol and centrifuged at 12,000 × g for 10 min. The absorbance of the supernatant was noted through a spectrophotometer (UV-3902, UNICO, MDN, USA) at 665, 663 and 475 nm wavelengths.

### Root morphology and anatomy

Roots were uprooted after 15 days of water treatments, cleaned and scanned through a scanner, and WinRHIZO Pro root analysis software (WinRHIZO 2003, Quebec, Canada) was used to determine the total root length, diameter, area, surface area, volume, and number of roots. Root cross-sections were obtained by uprooting the roots at the end of the experiment and repeatedly washing with tap water, and the segments were stored in FAA (formalin-acetic acid alcohol) solution. The distal 5 cm from the primary root of 6 plants from each treatment was selected and stored at 4 °C for 7–10 days until further processing. Freehand cross-sections were cut 3 cm from the distal region and then stained with Sudan 7b for one hour, washed with deionized water, and mounted on a slide with a solution of 70% glycerol. The cross-sections were placed under a light microscope (Olympus, Japan) with a magnification of 50 µm, and the measurements of cortex diameter and xylem vascular bundle were determined using the polygon tool of the software associated with the microscope.

### Antioxidant enzymes

#### Sample preparation

Leaf samples (0.2 g) were ground in liquid nitrogen, homogenized in 1.5 ml of 0.1 M phosphate buffer followed by 50 mg of insoluble PVP (polyvinylpyrrolidine) and 1 mM EDTA (pH 7.5) and centrifuged at 15,000 g for 20 min at 4 °C [30]. The supernatant was collected in NAP-5 columns for further use. Separate columns were used for the ascorbate peroxidase assay, where the desalting buffers consisted of 1 mM ascorbate. Proteins were quantified according to [31].

#### Catalase

Catalase activity was determined by adding 100 µL of sample supernatant, 20 µL of H_2_O_2_, 800 µL of 0.1 M phosphate buffer, and 1 mM EDTA (pH 7.5) in a quartz cuvette at room temperature [30]. The decrease in absorbance was observed using a spectrophotometer (U3902, USA) at 240 nm for 3 min.

#### Ascorbate peroxidase (APX)

Ascorbate peroxidase activity was assayed according to [30] using a microplate reader. Ascorbate (10 mM) was added to 50 µL desalted extract, 890 µL of 0.1 M phosphate buffer, and 1 mM EDTA (pH 7.5) in a quartz cuvette at room temperature. The reaction was started by adding 10 µL of 20 mM H_2_O_2_, and a decrease in absorbance was recorded at 290 nm for 2–3 min.

#### Dehydroascorbate reductase (DHAR)

DHAR activity was assayed according to [30]. A solution containing 50 µL of 4 mM DHA, 25 µL of 100 mM GSH, 905 µL of 0.1 M phosphate buffer, and 1 mM EDTA (pH 7.0) was added to a quartz cuvette. The reaction mixture was started by adding 20 µL of desalted extract. The decrease in absorbance was noted for 2–3 min at 265 nm wavelength.

#### Glutathione reductase (GR)

GR activity was determined according to [30], with 100 µL of the desalted extract added to 10 µL of 10 mM NADPH, 880 µL of 0.1 M phosphate buffer, and 1 mM EDTA (pH 7.5) in a glass cuvette at room temperature. The reaction was started by adding 10 µL of 50 mM GSSG, and the decrease in absorbance was noted at 340 nm for 2–3 min.

#### Monodehydroascorbate reductase (MDHAR)

Fresh leaf samples (0.2 g) were homogenized in liquid nitrogen, 50 mg of insoluble PVP was added, and then a freshly prepared mixture of 1 mL of 50 mM MES/KOH buffer (pH 6.0), 1 mM ascorbate, 40 mM KCl, and 2 mM CaCl_2_ was added. The mixture was centrifuged at 14,000 rpm at 4 °C for 10 min. The supernatant (50 µL extract) was used immediately for assay by adding 925 µL of 50 mM HEPES (pH 7.6), 10 µL of 25 mM NADPH, and 10 µL of 250 mM ascorbate. The reaction was started by adding 0.3 units of ascorbate oxidase (5 µL). The decrease in absorbance was noted for 2–3 min at 340 nm wavelength on a spectrophotometer [30].

### Photosynthetic measurements

The tomato plant net photosynthesis rate, stomatal conductance, intercellular CO2 concentration, and transpiration rate were observed through a portable photosynthetic system (LI-COR 6400XT) from 10:00 a.m. to 12:00 p.m. on a sunny day after 15 and 35 days of treatments.

### Extraction, purification, and quantification of phytohormones

Plant endogenous hormones auxin (IAA), abscisic acid (ABA), gibberellic acid (GA_3_), and cytokinin (CTK) were extracted and purified as described by [32]. Tomato leaves and roots (500 mg) were homogenized in liquid nitrogen, and an internal standard (25 µL each of d5-IAA (2 ng/μL), d6-ABA (0.25 ng/μL), d2-GA (2 ng/μL), and d5-ZT (0.25 ng/μL)) was added to each sample (Shanghai, Yuanye Bio-Technology Co., Ltd., Shanghai, P. R China). The extraction solvent (isopropanol: H_2_O: concentrated HCl = 2:1:0.002, v/v/v) at 0.5 ml was added to the samples and shaken and centrifuged for 30 min at 100 rpm and 4 °C. The supernatant was collected, and 1 mL of dichloromethane was added and shaken for 25 min. The solvent was concentrated by a nitrogen evaporator, and the dried residue was redissolved in 1 ml methanol. Afterward, the mixture was centrifuged again and the supernatant was transferred to vials for LC–MS analysis. The samples were quantified using LC–MS (Agilent 1260 HPLC system and an AB Qtrap 5500 triple quadrupole mass spectrometer with an electrospray ionization source) in MRM (multiple-reaction monitoring) mode using an Agilent SB-C18 column (50 × 4.6 mm, 1.8 μm) with a sample flow rate of 0.8 mL/min for the mobile phase. Each phytohormone was measured as described in a previous study [33].

### Soil enzymes

Soil pH (1:2), electrical conductivity (EC) and organic matter were measured according to [34]. The total nitrogen, phosphorus, potassium and total organic carbon were determined according to [35]. The soil particle size distribution (Mastersizer 2000E laser diffractometer, UK) was measured according to [36]. Soil enzymatic activities were determined after harvesting tomato plants at the end of the experiment. Soil samples were collected from ten random pots in each treatment in triplicate, and soil ß-glucosidase [37], alkaline phosphatase [38], and urease activities [39] were determined accordingly.

### Statistical analysis

The experiment was designed as a factorial design with water content as one factor and soil texture as another. The treatment means were subjected to two-factor ANOVA, and the means with significant differences at p ≤ 0.05 were separated using Tukey’s HSD test. Plant endogenous hormones (IAA, GA_3_, CTK, and ABA) were correlated with antioxidant enzymatic activities (APX, CAT, MDHAR, DHAR, and GR) in each interval via a CCA (canonical correspondence analysis) using Past 3.14.

## Results

### Plant length and chlorophyll pigments

Tomato plants grown in the different soil textures showed a significant difference in growth and chlorophyll contents with or without water deficit conditions. In the well-watered treatments, the highest plant length, chlorophyll b, and total chlorophyll (a + b) contents were observed in Yangling soil while the highest chlorophyll a and carotenoid contents were observed in Hanzhong soil. Water deficit conditions significantly reduced the plant length, chlorophyll content, and carotenoid content. As presented in Table 2, the lowest plant length was observed in Hanzhong soil, followed by Yulin soil while significant variations were not observed for the chlorophyll a, total chlorophyll content, and carotenoid contents among the soil textures under the water deficit conditions.

**Table 2:** Tomato plant length (cm) and chlorophyll and carotenoid contents (mg g^−1^ FW) under soil water contents of 75–70% and 55–50% in soils from Hanzhong, Yulin, and Yangling of Shaanxi, China

Soil	Treatments	Plant length (cm)	Chlorophyll a (mg g^−1^ FW)	Chlorophyll b (mg g^−1^ FW)	Chlorophyll a + b (mg g^−1^ FW)	Carotenoid (mg g^−1^ FW)
Hanzhong	Control	93.39 ± 1.57 b	23.25 ± 0.75 a	8.79 ± 0.35 ab	32.04 ± 0.19ab	6.18 ± 0.12 a
	Deficit	65.54 ± 2.14 d	19.88 ± 2.49abc	7.46 ± 0.68 b	27.34 ± 0.11 bcd	3.72 ± 0.08 c
Yulin	Control	97.98 ± 4.24 ab	21.29 ± 1.78 abc	9.82 ± 0.83 a	31.11 ± 0.15 abc	5.62 ± 0.07a
	Deficit	75.15 ± 3.75 c	17.27 ± 1.47 c	7.79 ± 0.18 b	25.09 ± 0.23 d	4.27 ± 0.13Bc
Yangling	Control	103.74 ± 2.63 a	22.89 ± 0.97 ab	10.12 ± 0.24 a	33.03 ± 0.18 a	5.59 ± 0.14 a
	Deficit	81.09 ± 1.72 c	18.14 ± 0.81 bc	7.94 ± 0.12 b	26.08 ± 0.17 cd	4.52 ± 0.09 b
Soil (S)		*	NS	*	NS	NS
Treatment (T)		*	*	*	*	*
Soil x Treatment (SXT)		*	NS	NS	NS	*

Treatment values presented for comparison are the means of three replications (± SE, standard error). Means followed by different letters are significantly different (*p* ≤ 0.05) according to Tukey’s test. *NS* not significant; *P < 0.05

### Tomato root growth attributes

According to Table 3, tomato root growth attributes showed significant differences when grown in soil with different textures and water contents. The total root length, total root area, total root volume, root diameter, and root number were significantly different in different soils. The highest root area, volume and number were observed in Yangling soil, while the highest root diameter was observed in Yulin soil under the well-watered conditions. Moreover, prominent differences were not observed in the root surface area. Water deficit conditions altered root morphological attributes in different soil textures. The highest total root length, area, and number under the water deficit conditions were observed in Yangling soil, while the highest total root volume and root diameter were observed in Yulin soil.

**Table 3:** Total root length (cm), area (cm^2^), surface area (cm^2^), root volume (cm^3^), root diameter (mm), and number for tomato grown under soil water contents of 75–70% and 55–50% and with different soil textures of China

Soil	Treatments	Total root length (cm)	Area (cm^2^)	Surface area (cm^2^)	Root volume (cm^3^)	Root diameter (mm)	Root number
Hanzhong	Control	844.7 ± 10.8 d	80.01 ± 7.9 bc	354.87 ± 16.5 abc	10.99 ± 1.1 de	0.91 ± 0.05 d	946 ± 26.3 bc
	Deficit	893.5 ± 22.7 c	65.83 ± 8.6 d	326.35 ± 12.8 c	7.81 ± 0.8 E	0.74 ± 0.08 e	858 ± 29.8 c
Yulin	Control	885.6 ± 6.23 cd	84.41 ± 10.9 ab	368.17 ± 14.8 a	21.93 ± 2.2 ab	1.26 ± 0.07 a	1013 ± 31.8 ab
	Deficit	1008.6 ± 17.5 b	72.87 ± 12.6 cd	333.37 ± 16.2 bc	17.67 ± 1.8 bc	1.15 ± 0.06 b	967 ± 17.8 bc
Yangling	Control	967.1 ± 14.3 b	91.75 ± 11.8 a	386.17 ± 11.5 a	24.16 ± 1.4 a	1.05 ± 0.05 c	1143 ± 46.8 a
	Deficit	1071.2 ± 22.7 a	77.97 ± 8.4bc	347.96 ± 12.4 bc	15.16 ± 1.1 cd	0.89 ± 0.08 d	1083 ± 28.7 ab
Soil (S)		*	*	*	*	*	*
Treatment (T)		*	*	*	*	*	*
Soil x Treatment (SXT)		*	NS	NS	NS	*	NS

Treatment values presented for comparison are the means of three replications (± SE, standard error). Means followed by different letters are significantly different (*p* ≤ 0.05) according to Tukey’s test. *NS* not significant; *P < 0.05

### Antioxidant enzymes

**Fig. 1:**
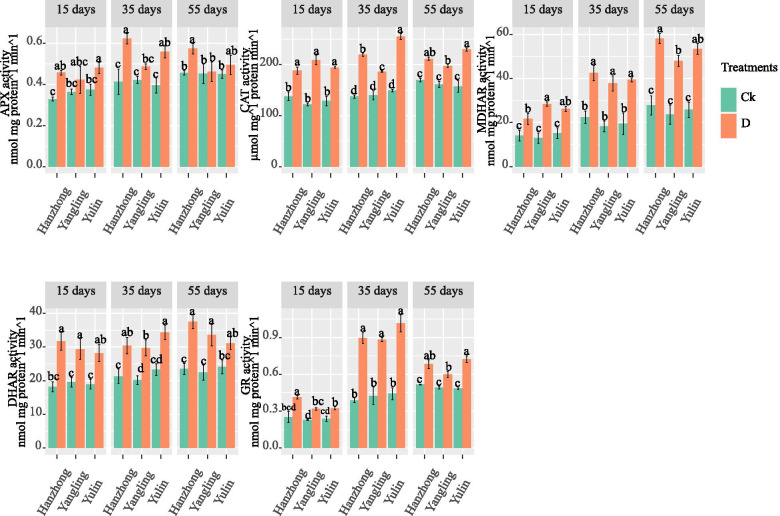
Ascorbate peroxidase (APX), monodehydrogenase reductase (MDHAR), dehydrooxidase (DHAR) (nmol mg protein^−1^ min^−1^), and catalase (CAT) activity (µmol mg^−1^, protein min^−1^) of tomato plants under soil water contents of 75–70% and 55–50% in the different soil textures of China. Treatment values presented for comparison are the means of three replications (± SE, standard error). Means followed by different letters are significantly different (*p* ≤ 0.05) according to Tukey’s test

As shown in Fig. 1, the antioxidant enzyme activities showed significant differences in different soil textures after 15, 35, and 55 days under the water deficit conditions. The overall highest APX activity was observed in plants grown in Hanzhong soil for 35 days under water deficit conditions. The CAT activity in the water deficit treatment was the highest in the plants grown in Yulin soil for 35 days and then subjected to 55 days of water deficit conditions. As shown in Fig. 1, the activity of MDHAR gradually increased after 15 days of water deficit conditions and was the highest in Hanzhong plants after 55 days. The overall highest activity of DHAR was observed in Hanzhong soil after 55 days, followed by Yulin soil after 35 days of water deficit conditions. The activity of GR drastically increased after 35 days of water deficit conditions in Yulin soil; however, its activity declined after 55 days. Moreover, there were no significant differences in antioxidant enzymatic activity under the well-watered treatment and different soil textures.

### Photosynthesis

**Fig. 2:**
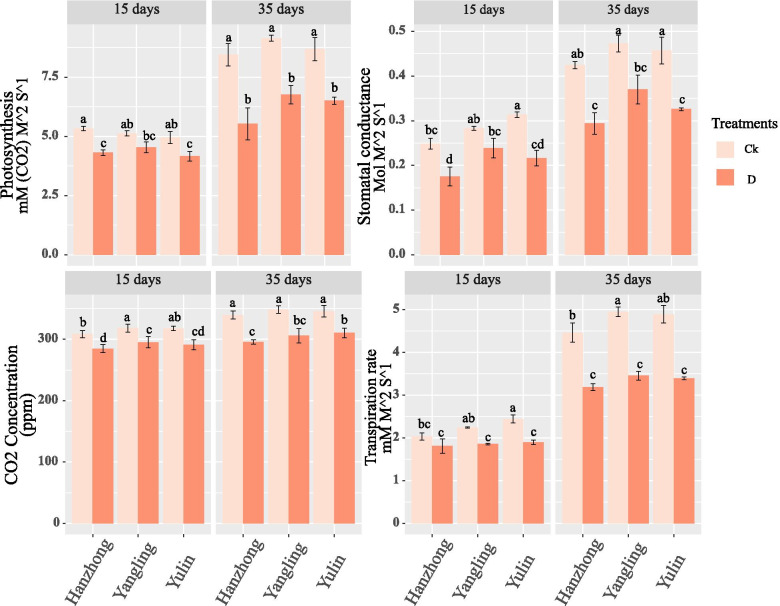
Photosynthesis (mM (CO_2_) M^−2^ S^−1^), stomatal conductance (mol M^−2^ S^−1^), carbon dioxide (ppm), and transpiration rate (mol M^−2^ S^−1^) of tomato plants grown under soil water contents of 75–70% and 55–50% and different soil textures in China. Treatment values presented for comparison are the means of three replications (± SE, standard error). Means followed by different letters are significantly different (*p* ≤ 0.05) according to Tukey’s test

Tomato gaseous exchange parameters showed differences under both different soil textures and water treatments (Fig. 2). The photosynthetic rate and stomatal conductance were significantly higher in Hanzhong and Yulin soils after 15 days in the well-watered treatments, while they were not significantly different after 35 days. On the other hand, the water deficit conditions decreased these attributes, and significant differences were not observed under the different soil textures. However, a slight difference in stomatal conductance was observed in Yangling soil after 15 days of treatment. The carbon dioxide exchange rate and transpiration rate were significantly higher in Yangling and Yulin soils, respectively, after 15 days of the well-watered treatment. The carbon dioxide exchange rate in the well-watered treatment did not show any significant differences after 35 days, while the transpiration rate was significantly higher after 35 days in Yulin soil. The water deficit conditions significantly decreased the carbon dioxide exchange rate and transpiration rate in different soil textures, however, the differences were not too obvious among different soil textures.

### Plant endogenous hormones

#### Gibberellic acid (GA_3_)

**Fig. 3:**
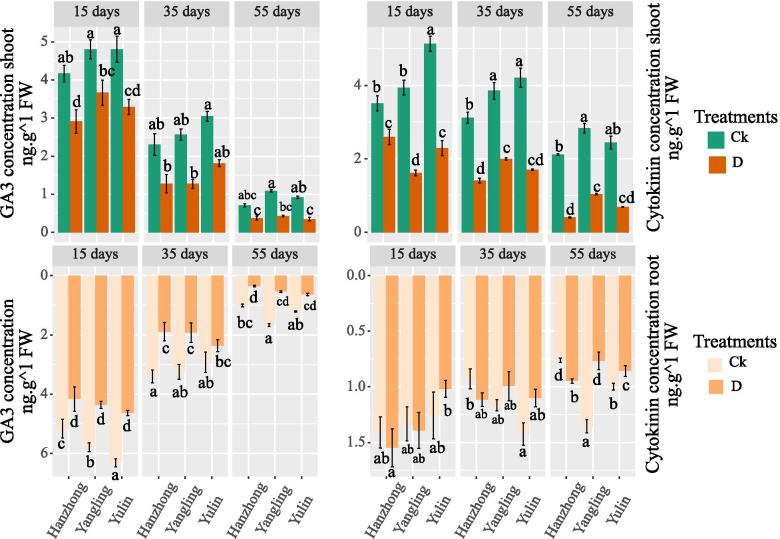
Gibberellic acid (GA_3_) and cytokinin content (CTK) (ng g^−1^ FW) in the leaves and roots of tomato plants under soil water contents of 75–70% and 55–50% in the different soil textures of China. Treatment values presented for comparison are the means of three replications (± SE, standard error). Means followed by different letters are significantly different (*p* ≤ 0.05) according to Tukey’s test

Tomato plant endogenous hormone values are presented in Fig. 3. The leaf GA_3_ content was not significantly different among the different soil textures after 15, 35, and 55 days under the well-watered treatment. However, there were significant differences after 15 and 55 days in the roots, with the highest values obtained in Yulin and Yangling soils, respectively. The GA3 content under the water deficit conditions was significantly decreased only in the leaves among different soil textures after 15 days of treatments; however, it was not significantly different in the leaves and roots among different soil textures after 35 and 55 days of treatments.

#### Cytokinin (CTK)

The CTK content in the leaves and roots showed a descending trend after 15 days in both water treatments (Fig. 3). The leaf CTK content showed significant differences among different soil textures under the well-watered treatments. The highest values were noted in Yulin soil after 15 and 35 days and in Yangling soil after 55 days. Similarly, the highest root CTK contents were observed in Yulin and Yangling soil after 35 and 55 days of the well-watered treatment. There was no significant difference observed in the root CTK content after 15 days of the well-watered treatment. The CTK content under limited water availability decreased significantly according to the soil texture in the well-watered treatments. The highest values were noted in Hanzhong soil after 15 days of treatment and in Yangling soil after 35 and 55 days of treatment. Moreover, Hanzhong soil showed maximum root values after 15 and 55 days under the water deficit conditions. There were no significant differences among soil textures after 35 days of limited water availability.

#### Auxin (IAA)

**Fig. 4:**
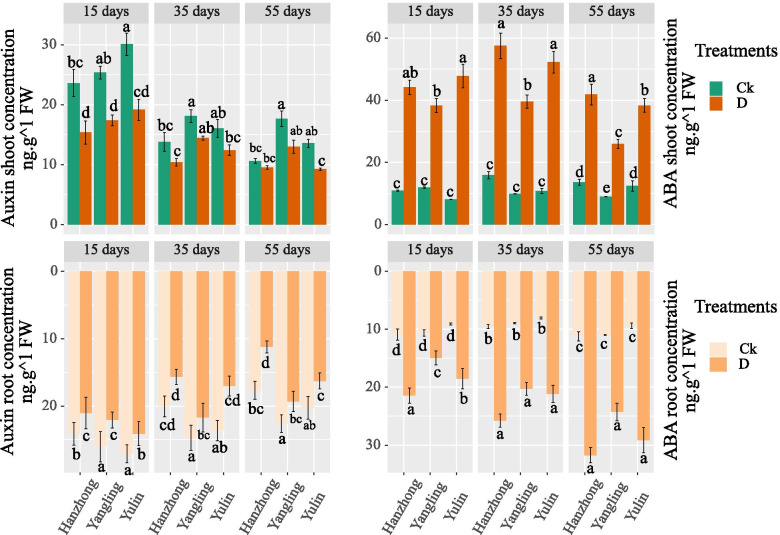
Auxin (IAA) and abscisic acid (ABA) contents (ng g^−1^ FW) in leaves and roots of tomato plants under soil water contents of 75–70% and 55–50% in the different soil textures of China. Treatment values presented for comparison are the means of three replications (± SE, standard error). Means followed by different letters are significantly different (*p* ≤ 0.05) according to Tukey’s test

The IAA content in the leaves and roots gradually decreased after 15 days in both water treatments (Fig. 4). The IAA content in the leaves showed significant differences among different soil textures under the well-watered treatments. The highest results were obtained in Yulin soil after 15 days and in Yangling soil after 35 and 55 days of treatment. A similar trend was also observed in the root IAA content, where the maximum values were recorded in Yulin soil after 15 days and in Yangling soil after 35 and 55 days of treatment. Limited water availability decreased the IAA content in both leaves and roots compared to their respective controls. Significant differences were obtained in Yangling soil after 35 days in the water deficit treatment, while there were no significant differences after 15 and 55 days. Moreover, there were obvious differences in the root auxin content of different soil textures. The highest results were recorded in Yulin soil after 15 days and in Yangling soil after 35 and 55 days of limited water availability.

#### Abscisic acid (ABA)

The ABA content in leaves showed a significant difference among soil textures in Hanzhong soil after 15 days of the well-watered treatment. There were no significant differences in the ABA contents in the leaves and roots among the different soil textures under the well-watered treatment (Fig. 4). However, the ABA content in the leaves and roots escalated after every interval under limited water availability. The highest ABA content in leaves was recorded in Yulin soil after 15 days and in Hanzhong soil after 35 and 55 days of treatments. Moreover, the ABA content in roots showed significantly higher results in Hanzhong soil after 15 and 55 days; however, significant differences were not observed among the soil textures was observed after 35 days of limited water availability.

### Root anatomy

**Fig. 5:**
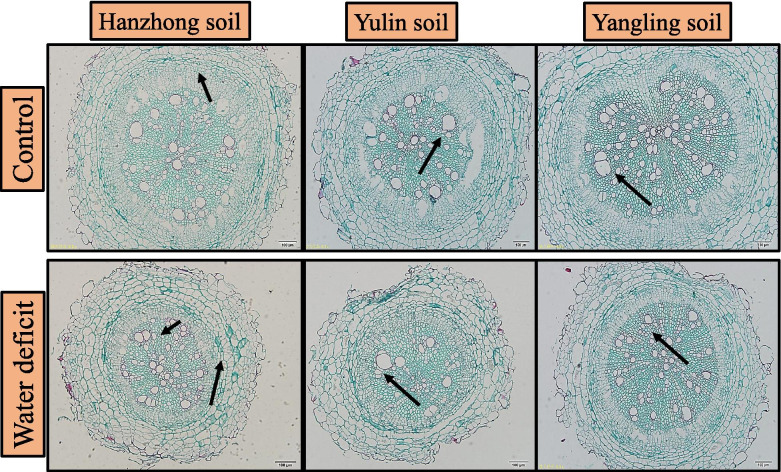
Taproot cross-sections of tomato plants under soil water contents of 75–70% (control) and 55–50% (water deficit) in the soils of Hanzhong, Yulin, and Yangling in Shanxi, China

**Fig. 6:**
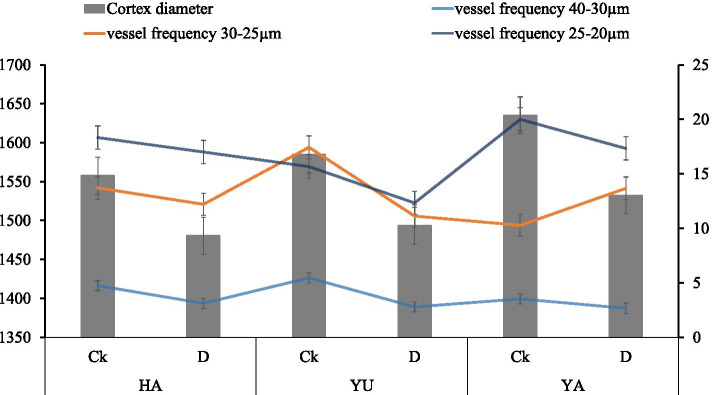
Taproot cortex diameter and xylem vessel frequency of tomato roots under soil water contents of 75–70% (Ck) and 55–50% (D) of the soils of Hanzhong, Yulin, and Yangling in Shaanxi, China, respectively. Treatment values presented for comparison are the means of three replications (± SE, standard error). Means followed by different letters are significantly different (*p* ≤ 0.05) according to Tukey’s test. HA, Hanzhong; YU, Yulin; and YA, Yangling

The root anatomy showed differences under the water deficit conditions and soil textures. The highest root cortex diameter was observed in Yangling soil and then Yulin soil in the well-watered treatments. Significant differences were not observed under the water deficit conditions among different soil textures; however, the lowest cortex diameter was noted in Hanzhong soil. The total area and distribution of xylem vessel diameter decreased under the water deficit conditions. The highest xylem vessel diameters, which ranged from 40–30 µm and 30–20 µm, were observed in Yulin soil and a 25–20-µm diameter was observed in Yangling soil under the well-watered conditions; however, the area and diameter were significantly decreased under the water deficit conditions. As shown in Figs. 5 and 6, the distribution of xylem vessel diameters was significantly reduced under the water deficit conditions. The highest values under the water deficit conditions were observed in Hanzhong soil, followed by Yangling soil. The results were statistically significant in the distribution of xylem vessel diameters of 30–25 µm and 25–20 µm in Yulin and Yangling soils, respectively, under the water deficit conditions; however, the differences among different soil textures were not statistically significant at p ≤ 0.05.

### Soil enzymatic activities

**Fig. 7:**
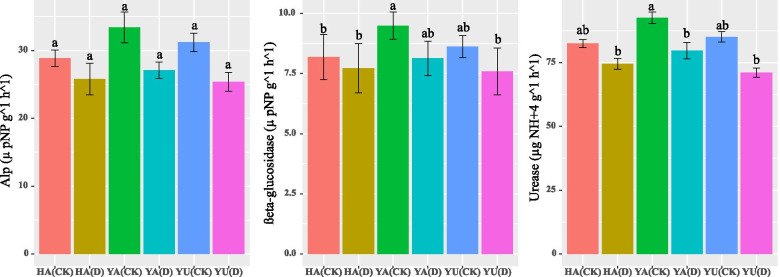
Soil urease (µg NH^+^4 g^−1^ h^−1^), ß-glucosidase (µ pNP g^−1^ h^−1^) and alkaline phosphatase [(Alp) µpNP g^−1^ h^−1^] activity under soil water contents of 75–70% (CK) and 55–50% (D) in the soils of Hanzhong, Yulin, and Yangling in Shaanxi, China. Treatment values presented for comparison are the means of three replications (± SE, standard error). Means followed by different letters are significantly different (*p* ≤ 0.05) according to Tukey’s test. HA, Hanzhong; YU, Yulin; and YA, Yangling

Soil enzymatic, ß-glucosidase and urease activities showed significant differences among different soil textures under the well-watered treatments, while significant differences were not observed under the water deficit conditions (Fig. 7). The highest ß-glucosidase activity was recorded in Yangling soil, followed by Yulin soil, and the highest urease activity was noted in Yangling soil, followed by Hanzhong soil. Significant differences were not observed in alkaline phosphatase activity among the different soil textures and water treatments.

### CCA (canonical correspondence analysis) of endogenous hormones and antioxidant enzymes

**Fig. 8:**
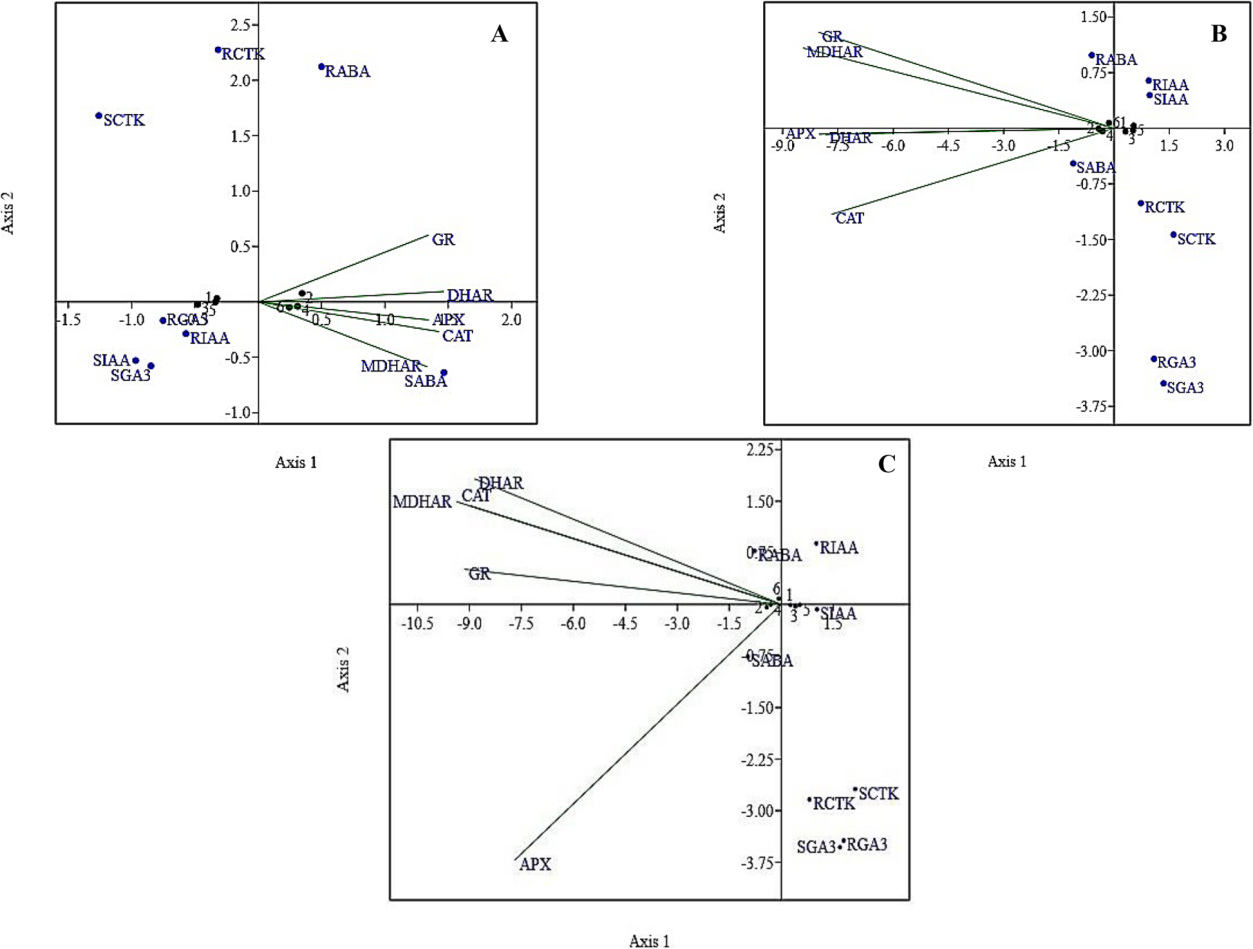
CCA (canonical correspondence analysis) of endogenous hormones and antioxidant enzymes **a** after 15 days, **b** 35 days, and **c** 55 days of water deficit conditions. APX = ascorbate peroxidase; CAT = catalase; monodehydrogenase hydroascorbate reductase = MDHAR; dehydroascorbate reductase = DHAR; glutathione reductase = GR; gibberellic acid in the leaves = SGA3 and roots = RGA3; cytokinin levels in leaves = SCTK and roots = RCTK; auxin content in the leaves = SIAA and roots = RIAA; abscisic acid in the leaves = SABA and roots = RABA

The CCA results (Fig. 8) show that the plant endogenous hormone ABA concentrations in the roots and leaves were correlated with the GR and DHAR activities after 15 days of water deficit conditions (Fig. 8a); however, the IAA and CTK contents in the leaves and roots were correlated with the increase in GR, APX, and MDHAR activity after 35 days of water deficiency (Fig. 8b). Moreover, after 55 days of water deficit conditions (Fig. 8c), the IAA, CTK, and GA_3_ contents were highly correlated with the activity of antioxidant enzymes (DHAR, CAT, MDHAR, and GR), which ultimately led to a decrease in ABA content.

## Discussion

Tomato plant growth and development are highly dependent upon the soil water availability and type. Studies have thus far determined the responses of plants to abiotic stresses; however, in our work, we determined the root responses under different soil textures and limited water availability. Our results show that plant height, root architecture, and chlorophyll pigments were affected by different soil textures and water availabilities. Plant roots undergo various morphological changes under different soil textures and water contents [18]. A compact soil texture consists of a higher percentage of clay particles that are in close contact with each other and possess fewer void spaces, thus increasing its bulk density [40]. The compaction of soil modifies the physical properties of the soil (bulk density and soil porosity), which further influence its chemical properties, faunal diversity, and plant growth [40–42]. Compact soil restricts root growth and causes an imbalance in the root shoot relationship by consuming more of its energy in producing new roots [43]. In our results, the root length and diameter decreased in Hanzhong soil while the root area, diameter, root number, and root volume were higher in Yangling soil. Restricted root growth further predisposes plants to drought stress, with a large reservoir of water and nutrients going unexplored. Under these circumstances, the roots undergo adaptive measures by changes in their anatomy and root architecture system to avoid or tolerate these unfavourable environmental conditions [44]. Water deficit conditions negatively affect root growth attributes and leaf chlorophyll pigments. The decrease in shoot and root attributes might be associated with the significant amount of energy being served by the roots when they do not search for water and nutrients [45]. Compact soil simultaneously possesses an additional obstacle to the roots, thus placing plants under stress conditions [46]. The higher carotenoid contents in Yangling soil might be related to the scavenging of O_2_^−1^, decreased production of ROS by chlorophyll and protection of the photosynthetic apparatus [47, 48]. Root growth under mild water deficiency might be impaired, which alters source productivity; however, according to [49], the chlorophyll pigments and photosynthesis performance are sometimes preserved under stress conditions. Water deficit conditions delimit mitosis and cell elongation due to the loss of turgor pressure [50]. The decreased cell size and retarded growth might be associated with the irreversible changes in anatomy over time [51].

Under unfavorable environmental conditions, roots adopt various adaptations, including changes in anatomy that can lead to differences in hydraulic function. Xylem vessels are associated with the root system and allow the plant to meet the source-sink water demand, and they are highly altered under abiotic stress conditions [52]. In our study, the decrease in total xylem vessel area and root cortex diameter under the water deficit conditions may lead to resistance in water transport. The lower total xylem area in the dry treatments may also prevent plants from embolizing with increasing xylem water tension [53]. These changes in xylem vessels are irreversible, highly affect plant water requirements and reduce growth [52], and they can only be overcome by growing new roots and increasing the water availability to balance the water tensions in the xylem. The crosstalk of hormones and balance of redox reactions may lead to more carbon allocation to the roots to increase the number and surface area of roots to compensate for the changes in root hydraulic functions.

In our results, the carbon dioxide concentration, stomatal conductance, and transpiration rate were altered under different soil textures and soil water contents. Low turgor pressure causes the closure of stomatal openings, and due to diffusion limitations, a decrease in pressure occurs, which results in photochemical restrictions and impairs photosynthetic machinery [54, 55]. The photosynthesis machinery is a well-established source of ROS in plants. The regulatory system of plants to restrict the overproduction of ROS is mainly dependent upon root water uptake [56]. The water molecules in the higher soil bulk density adhere to the soil particles and occupy soil pores, thus decreasing their air circulation [46]. Decreases in the water infiltration rate and air circulation may lead to anoxic conditions and severely decrease root hydraulic activity. Our results were similar to those of [54], who stated that the closure of stomata is highly sensitive to the soil moisture content because it responds to root-to-shoot signaling of ABA, whose production is triggered under unfavorable conditions. The lower photosynthesis rate under the water deficit conditions might be due to the reduced accumulation of photosynthetic electron transport components and enzymes involved in photosynthesis, which potentially reduce molecular oxygen and result in the production of ROS [46]. The synthesis of the Rubisco enzyme is restricted by the binding of inhibitors, such as 2-carboxyaribinitol 1-phosphate, to the catalytic site of Rubisco. The carboxylation of Rubisco also declines due to the increase in the synthesis of ribulose 1,5-bisphosphate, which acts more as an oxygenase than carboxylase [57, 58]. Furthermore, the lower growth and photosynthetic machinery functions under the water deficit conditions are due to an imbalance of ROS production and antioxidant defence systems, which leads to oxidative stress in membranes, lipids, and proteins.

According to our results, the antioxidant enzymes were increased in Hanzhong soil at the onset of water deficit conditions, while their activity was lower compared to that of the other soils at later periods. The higher antioxidant defence system at the onset of water deficit conditions might be due to the lower root number, root area, and root volume resulting in a lower rhizosphere for the uptake of water and nutrients [52, 54]. Water deficiency mimics drought conditions and is accompanied by the overproduction of ROS (O_2_^−1^, OH^−^, O_2_^−^, H_2_O_2_), which react with plant cells, proteins, lipids, etc. and cause oxidative damage to plants. These ROS are scavenged by plant antioxidant enzymes, resulting in reduced damage to cells and even the death of cells [5, 59]. Antioxidant enzymes are the key elements of the plant defence system and participate in the detoxification of ROS produced under stress conditions. Several antioxidant enzymes have different levels of affinity for ROS and convert O_2_^−1^, O_2_^−^, OH^−^, etc. to oxygen and water [8]. In our results, antioxidant enzymatic activity was higher in Yulin soil as the water deficit conditions progressed. The CAT enzyme usually scavenges H_2_O_2_ and reduces the toxic levels of ROS [7]. Yulin soil has a higher content of sand and silt, and the available water either leaches quickly or evaporates, which may be the cause of an increase in O_2_^−1^ and H_2_O_2_ production [46]. Studies have revealed that an important ascorbate–glutathione cycle (APX, DHAR, MDHAR, and GR) present in the cytosol, mitochondria, and stroma of chloroplasts is crucial for scavenging O_2_^−1^, H_2_O_2_, and OH^−^ ions and plays a major role in maintaining the reduced glutathione pool [60, 61]. Compared with CAT, the APX enzyme catalyses the removal of H_2_O_2_ by using ascorbate as a reductant and is regenerated or synthesized through the MDHA or DHA pathway. Several studies have revealed that APX activity is increased under various biotic and abiotic stresses [21, 30]. In our results, APX activity was increased in Hanzhong soil under continuous water deficit conditions. The roots in compact soil may have a limited rhizosphere to explore water and nutrients, and water deficit conditions may lead to higher stress conditions compared with that observed for roots in soils with a lower bulk density [62]. Similarly, DHAR, with the contribution of oxidizing glutathione, catalyses the reduction of DHA to AsA to balance the ascorbate redox state [63]. MDHAR is associated with the ascorbate glutathione cycle and represents a major component in balancing the ascorbate redox state, and its activity increases with the increase in the AsA pool. The AsA pool is further consumed by APX as a reducing agent to scavenge hydrogen peroxide and produce water [63, 64]. Glutathione oxidized during the reaction of DHA in the regeneration of AsA is further reduced through NADPH- and NADP-dependent GR to complete the ascorbate glutathione cycle and continue scavenging of H_2_O_2_ by APX [65]. The higher activity of DHAR and MDHAR in Hanzhong soil may suggest the higher production of ROS due to water deficit conditions.

Under stress conditions, stress-responsive genes are expressed in plants and largely controlled by transcription factors, and they are subjected to very intricate regulation of phytohormones. In our results, the contents of plant endogenous hormones, such as IAA, CTK, and GA_3_ acid, in the shoots and roots declined as the water deficit conditions progressed. However, the ABA concentration increased to some extent and then declined under continuous water deficit conditions. The difference in concentration of ABA among the different soil textures can be related to the differences in soil porosity and bulk density. Restricted root growth leads to poor root-soil contact and ultimately limits the ability of the plant to absorb water and nutrients [46]. The lower auxin, GA3, and CTK concentrations in Hanzhong soil might be related to altered root growth due to soil compaction, and according to [66], if the soil is already suffering from degradation or drought conditions, then the drastic effects of soil compaction might double. Upon exposure to drought stress, plants trigger the synthesis of ABA, which has a major role in the adaptation of plants to stress conditions through stomatal closure and reduced growth [54, 67]. Moreover, ABA also induces certain genes that influence a variety of stress-countering factors to detoxify reactive oxygen species, such as transcription factors, protein transporters and enzymes for compatible solute metabolism and phospholipid signalling [68, 69]. The decrease in GA_3_, IAA, and CTK under drought conditions might be due to the inadvertent closure of stomata, which causes a reduction in carbon gain and affects plant development [70]. The higher growth and CTK concentration of plants grown in Yangling soil compared to Hanzhong soil in our work might be associated with the major role of CTK in delaying premature leaf senescence, which was also observed by [71]. Drought-induced leaf senescence usually coincides with decreasing endogenous CTK levels; however, studies have revealed that other factors, including antioxidant profiles or altered source-sink relationships, may also be responsible for senescence in plants [72]. The canonical correspondence analysis (Fig. 8) also showed the correlation of endogenous hormones in shoots and roots with antioxidant enzymatic activity. The decrease in IAA concentration can be attributed to the decrease in the growth of plants because IAA is synthesized in the rapidly dividing cells and tissues of the shoot and root meristem. Moreover, IAA degradation and conjugation occur due to the presence of oxidizing ROS agents, which disrupt the transport and distribution of IAA through relocation of PIN proteins (responsible for auxin transport) [73]. Similar results were also obtained by [13], who studied the role of auxin in delaying leaf senescence by using AUXIN RESPONSE FACTOR 1 (ARF1) and (ARF2) mutants and suggested that leaf senescence was positively regulated due to the repression of IAA signalling by ARF transcription factors. Moreover, the crosstalk of H_2_O_2_ and IAA signalling mediation were also correlated with the changes in glutathione redox status in a hyponastic phenotype of an *Arabidopsis* CATALASE2 mutant [13]. GA_3_ is typically known to respond antagonistically to ABA and has a limited role in stomatal conductance; however, it is involved in multiple signalling pathways and cross-talk among other hormones under drought stress [74]. GA concentrations were mediated in marigold due to the higher activity of SOD (superoxide dismutase) under drought stress [75]. The ameliorative role is attributed to the higher antioxidant activity, which promotes scavenging of ROS, and the involvement of WRKY transcription factors associated with the GA signalling pathway [76]. Moreover, the upregulation of xyloglucan endotransglycosylase genes (XET1 and XET1) and expansin genes (EXPB4 and EXPA4) may also maintain cell division and elongation in *Festuca arundinacea* under drought stress [77].

Debris from plants and microbes with carbon biomacromolecules is recycled through various enzymatic and catalytic processes in the soil by a consortium of microorganisms. This process consists of a myriad of complex biochemical reactions and transformation and decomposition of organic matter, humus synthesis, and their activity expresses the metabolic status of the soil [22]. In our work, the soil enzyme activity did not show any significant differences under the different soil water contents. However, a slight difference in ß-glucosidase and urease activity was observed among the different soil textures. The difference in soil enzymatic activities might be due to the lower nutritional status, organic matter content, and pH in the different-textured soils. In our results, Yulin soil showed a decrease in urease activity, which might be due to the lower clay particles in the soil. According to [78], soil urease activity is highly correlated with soil organic matter content, cation exchange, and clay particles. ß-glucosidase releases low molecular weight sugars, which are an energy source for soil microbes and play a vital role in the global C cycle [25]. The higher soil enzymatic activities in Yangling soil can be attributed to the higher soil organic matter content, nutritional status, and microbial activity, as documented by [26, 79, 80].

## Conclusions

Our findings indicate that soil texture and water deficit conditions have an important role in tomato plant root growth and development. The current findings suggest that soil compaction has a major role in the root architecture, growth and development. The Hanzhong soil (clay loam – clay) consisted of higher clay particles, which in turn restricted tomato shoot and root growth, physiology and overall plant development, which was more obvious under the water deficit conditions. The restricted root growth and altered source sink relationship caused an imbalance in hormonal crosstalk, which resulted in higher activities of ascorbate–glutathione cycle and ABA concentrations compared to Yangling and Yulin soil. The improved root growth attributes and hormonal crosstalk in Yangling (loam-clay loam) and Yulin soil (silt-sandy loam) can be attributed to higher silt and sand particles, which increase the soil porosity and improve the root growth architecture. The higher concentrations of IAA, GA_3_ and CTK in the roots of Yangling and Yulin soils with and without water treatments further reveal the role of soil texture in the improved growth and development of tomato plants. Soil enzymatic activities are highly dependent upon soil organic matter, nutrients, and clay particles; however, with increases in the clay particles in soil, the compaction of the soil increased. Further studies will be carried out to study the root hair sizes, their interaction with different soil textures, and their effect on the whole-plant hydraulic activity.

## Data Availability

The datasets used and/or analyzed during the current study available from the corresponding author on reasonable request.

## Abbreviations

ROS: Reactive oxygen species
O_2_: Oxygen radical
OH: Hydroxyl ion
H_2_O_2_: Hydrogen Peroxide
IAA: Auxin
CTK: Cytokinin
ABA: Abscisic acid
GA_3_: Gibberellic acid
APX: Ascorbate peroxidase
CAT: Catalase
DHAR: Dehydroascorbate reductase
MDHAR: Monodehydrogenase hydroascorbate reductase
GR: Glutathione reductase
